# Fin-ally together: the first UK zebrafish meeting

**DOI:** 10.1242/bio.062372

**Published:** 2026-01-07

**Authors:** Min-Kyeung Choi, Ekaterina Dvorianinova, Stephanie M. E. Jones, David Maley, Gemma Sutton, Yu Hsuan Carol Yang

**Affiliations:** ^1^ Living Systems Institute, University of Exeter, Exeter EX4 4QD, UK; ^2^ Department of Clinical and Biomedical Sciences, University of Exeter, Exeter EX1 2LU, UK; ^3^ Department of Biosciences, University of Exeter, Exeter EX4 4QD, UK; ^4^ Aquatic Resources Centre, University of Exeter, Exeter EX4 4QD, UK

**Keywords:** Zebrafish, Developmental biology, Human disease modelling, Toxicology, Neuroscience, Technology development

## Abstract

The inaugural UK Zebrafish Meeting 2025 (UKZF2025) hosted by the Living Systems Institute (LSI) at the University of Exeter, was held from 10 to 12 September 2025 in Exmouth and Exeter. The organising committee comprised LSI Group Leaders Steffen Scholpp, Soojin Ryu, Nikolas Nikolaou and Yu Hsuan Carol Yang, and the Facility Manager of Exeter's Aquatic Resources Centre, Greg Paull. The event brought together over 150 zebrafish researchers from across the UK to present their latest discoveries, share resources and expertise, and discuss strategies to strengthen the UK zebrafish research community. This Meeting Review highlights the key scientific themes showcased during the meeting, including developmental biology, human disease modelling, toxicology, neuroscience, technology development and alternative fish models. Furthermore, it provides a summary of the community session led by zebrafish facility managers from across the UK, emphasising potential opportunities for enhancing collaboration and resource sharing within the community.

## Teaser

**Summary:** The inaugural UK Zebrafish Meeting 2025 brought together over 150 UK researchers to share scientific advances and discuss strategies for strengthening the national zebrafish research community.

## Introduction

**Fig. 1.**
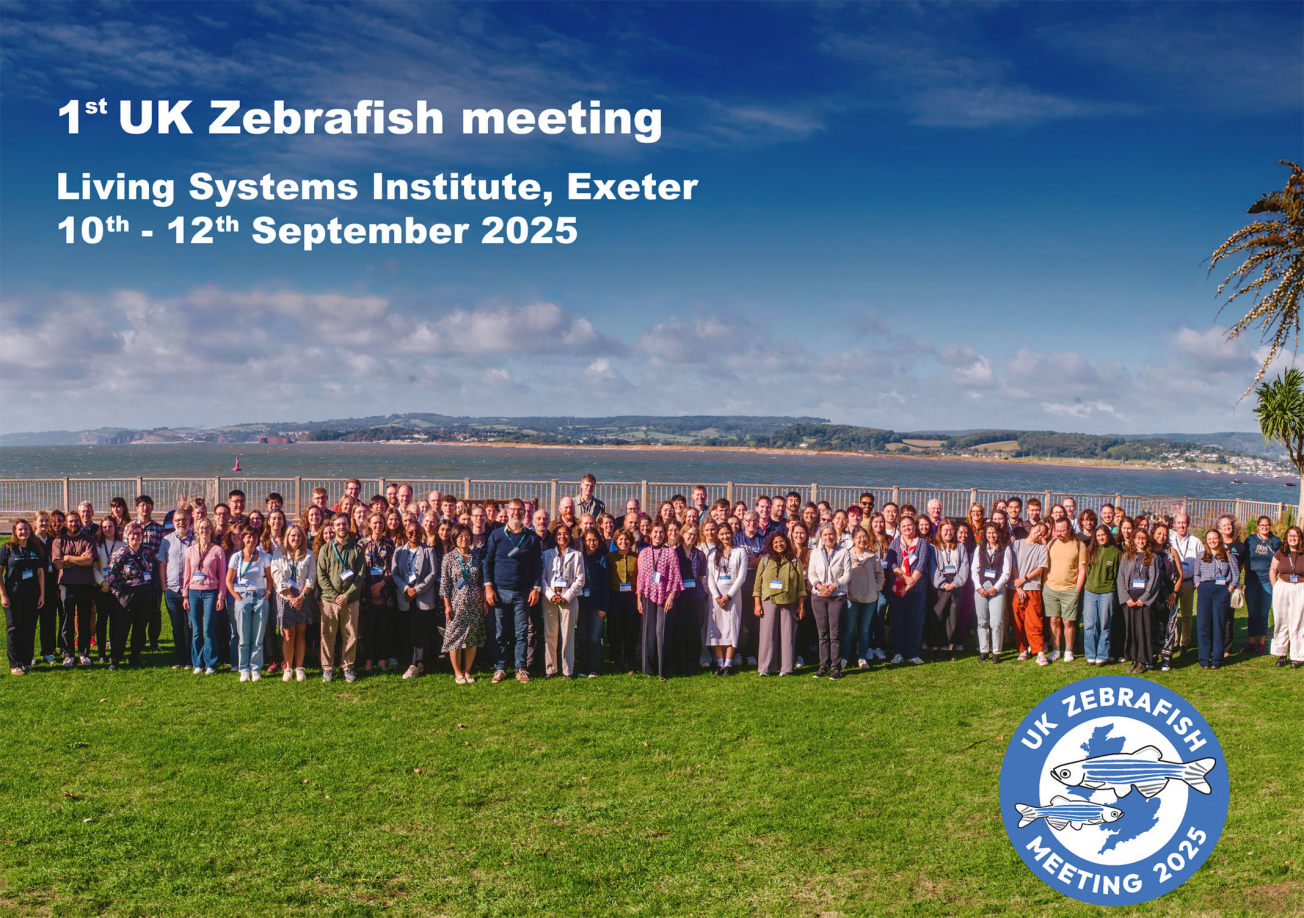
Group photo from UKZF2025, hosted by the Living Systems Institute at the University of Exeter.

**Fig. 2.**
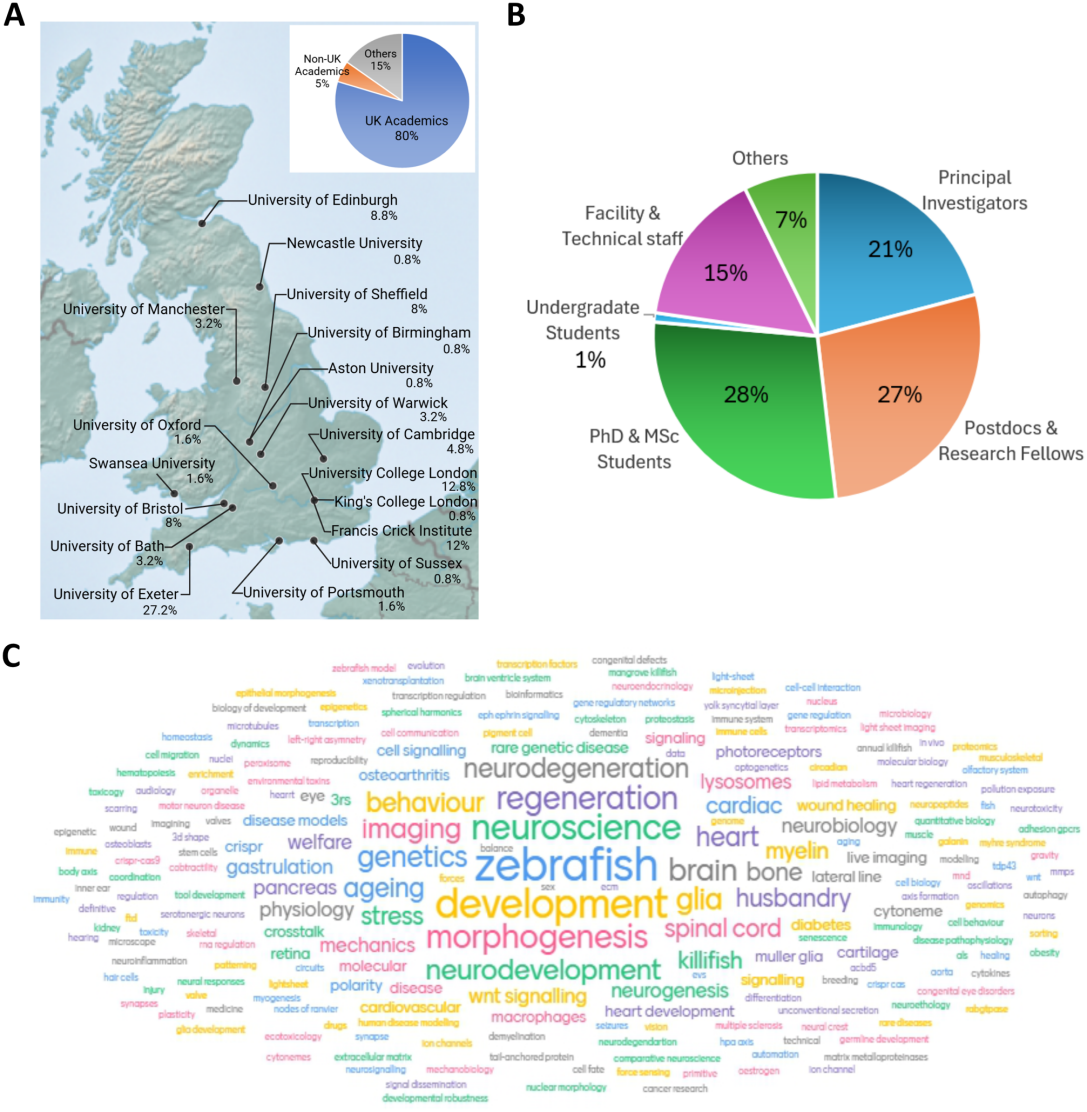
**Delegate diversity is represented by institutions, career stages and research interests.** (A) The majority of the delegates are working in UK academic institutions. The percentage of UK-based delegates from each institution are mapped to showcase the geographical diversity. (B) The percentage of delegates from different career stages highlights the strong representation of early-career researchers and facility staff. (C) A word cloud showcasing the diverse research interests from the community. The delegates were asked to describe their research interests using up to five keywords (generated using Mentimeter).

**Fig. 3.**
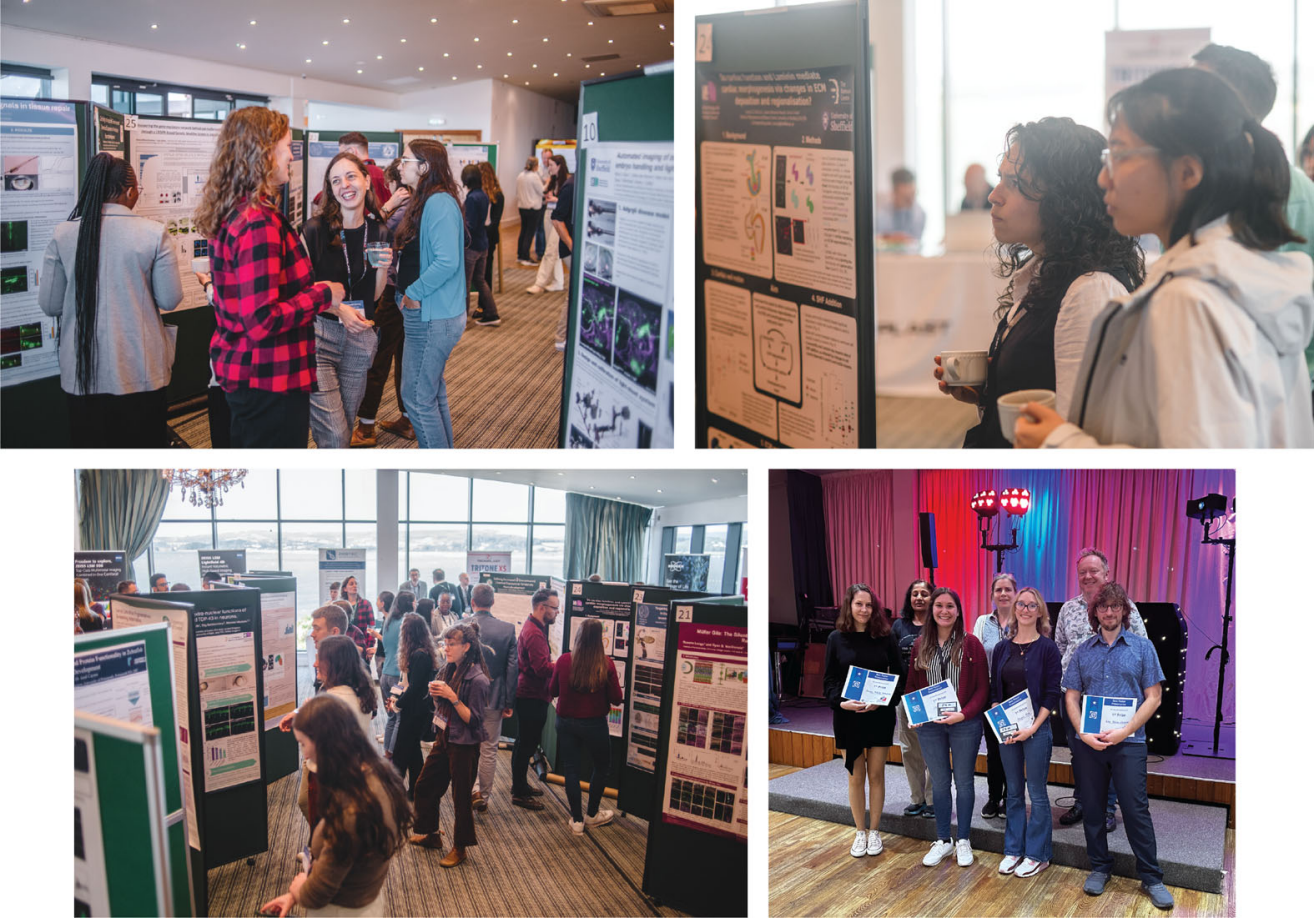
**Poster sessions and prize winners at UKZF2025.** Three prizes were awarded to early-career researchers for their poster presentation. Abigail Kite (Jordi Cayuso laboratory, University of Portsmouth) won the MSc poster prize for her study on the role of Mmp16 in neural development. Laura Bader (Rashmi Priya laboratory, The Francis Crick Institute) won the PhD poster prize for her work on nuclear homeostasis in cardiomyocytes experiencing mechanical deformations. Simona Mikula Mrstakova and Aitor Bañón-González (Rodrigo Young laboratory, UCL) won the postdoctoral poster prize for their joint poster on the study of the genetic basis of anophthalmia and microphthalmia using a CRISPR-based genetic screen in a sensitised zebrafish mutant background.

Researchers from across 18 UK academic institutions and four non-UK academic institutions attended the UK Zebrafish Meeting 2025 (UKZF2025) (Fig. 1 and Fig. 2A). The meeting was well attended by early-career researchers (Fig. 2B) and provided postdocs and PhD/MSc students a platform to network with the community and establish new collaborations. The meeting highlighted the diversity of zebrafish research across the UK (Fig. 2C), representing 40 different research groups, whose work was showcased in the short oral presentation sessions and during the poster sessions (Fig. 3). Although this Meeting Review only captures a subset of the cutting-edge research presented at UKZF2025, we aim to highlight the diversity of research interests, spanning six key interconnected themes: developmental biology, human disease modelling, neuroscience, toxicology, technology development and alternative fish models.

**Fig. 4.**
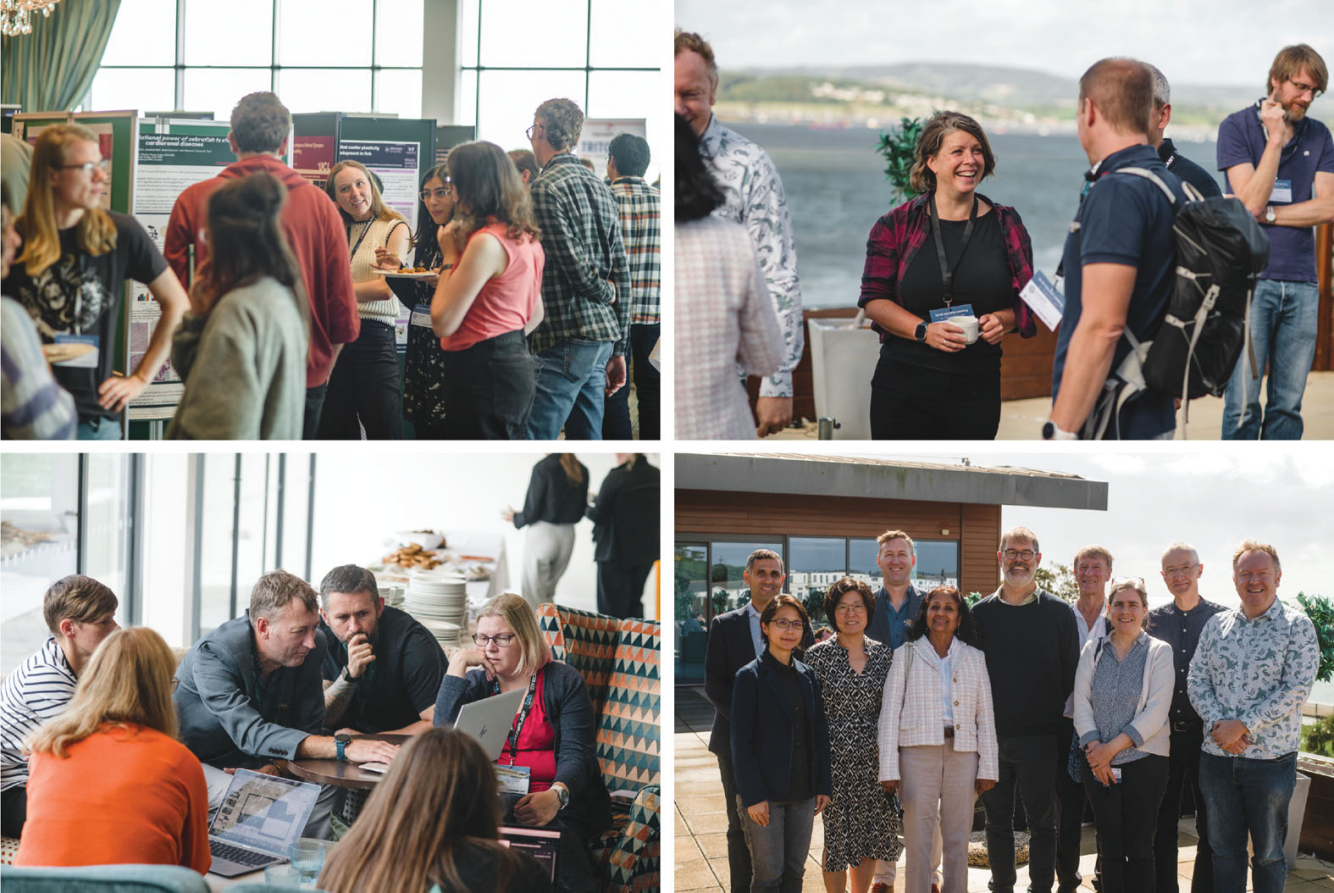
Snapshots of the UKZF2025 meeting in Exmouth.

A major accomplishment of bringing the community together in person was the emergence of synergies across research areas, which offers new opportunities to enhance research impact (Fig. 4). Accelerating these collaborations and opportunities for sharing of resources and knowledge transfer will require inter-institutional strategies and strong engagement with facility managers and staff, who were well represented at the meeting (Fig. 2B). By hosting the inaugural meeting and drawing on the combined strength of the multiple zebrafish groups at the Living Systems Institute and the state-of-the-art infrastructure of the Aquatic Resources Centre, Exeter is emerging as a key coordinating centre for the UK zebrafish network. During the community session, zebrafish facility managers presented the resources available across four different facilities and highlighted the services that can be offered to researchers beyond their own institutions. Overall, UKZF2025 was a springboard to strengthen the connections among UK zebrafish researchers at all career stages and to empower the delegates to explore new collaborative opportunities.

## Using zebrafish to understand the principles of development

Zebrafish emerged as one of the prominent model organisms in developmental biology over 25 years ago, and this meeting proved that it is still an exciting time to be a developmental biologist studying zebrafish embryogenesis. During the research talk sessions, speakers demonstrated their pioneering work using the latest technology in fluorescent reporters, optogenetics, imaging and genome editing. Some of the highlights included a talk from Scott Wilcockson (Caroline Hill laboratory, The Francis Crick Institute) on an Erk-specific signalling biosensor that has led their team to develop a new model of endoderm and mesoderm specification in the gastrulating zebrafish (Wilcockson et al., 2023). Clare Buckley (University of Manchester) presented a talk on force propagation in the zebrafish neural rod, with a novel optogenetic tool to manipulate cell contractility in a reversible manner in the live zebrafish embryo (Crellin et al., 2024 preprint). Tanya Whitfield (University of Sheffield) presented collaborative work identifying rare cell types in the olfactory epithelium: olfactory ionocytes and olfactory rod cells. The latter were initially characterised owing to the presence of a single 10 µm-long actin-rich apical projection (Cheung et al., 2021; Peloggia et al., 2025).

Finally, the first UK zebrafish meeting featured keynote talks from two well-renowned UK-based developmental biologists, Karuna Sampath (University of Warwick) and Rashmi Priya (The Francis Crick Institute). Karuna Sampath presented a fascinating story of her laboratory’s work dissecting the mechanisms controlling organisation of the zebrafish oocyte. Rashmi Priya presented her laboratory’s work untangling the process of zebrafish heart development featuring spectacular live imaging.

Together, these studies illustrate how developmental biology continues to drive innovation in the zebrafish field, setting the stage for its growing use in disease and translational research.

## Harnessing the power of zebrafish to tackle human diseases

Zebrafish have traditionally been an appealing organism for studying various human health conditions from neurodevelopmental disorders to cancer. Growing interest in using zebrafish in disease was reflected in the diversity of research talks given at the meeting. Becky Yarwood (Martin Lowe laboratory, University of Manchester) demonstrated how zebrafish can be used as a model to study hereditary spastic paraplegia type 82 – unlike mice, fish Pcyt2 mutants are viable during early development and share phenotypical similarities with patients. Stefan Schulte-Merker (University of Münster) shared a new model that allows the study of lymphangiogenesis, possibly shedding light on pathogenesis of diseases such as lymphedema (Uphoff et al., 2025).

Continuing with the topic of cardiovascular malformations, Chris Derrick (Newcastle University) talked about the potential use of zebrafish for investigating the causes of bicuspid aortic valve, the most common congenital heart disease. He showed that the mechanisms of arterial valve formation in zebrafish are well conserved between humans, mice and fish, despite differences in valve morphology (Derrick et al., 2025). Rodrigo Young [University College London (UCL) Institute of Ophthalmology] discussed how CRISPR modifier screens in zebrafish can be a powerful tool for identifying functions of congenital eye defect-related genes, highlighting the role of eye growth compensation in masking the effects of mutations that would otherwise result in an eye phenotype.

Jeremie Zappia (Chrissy Hammond laboratory, University of Bristol) explained how fractures in caudal fin ray bones may serve as a model to elucidate the role of neutrophils in haematoma and bone callus formation and how their pro-reparative role is involved in bone repair. Overall, the meeting presentations and posters covered a wide range of models addressing various human diseases, proving the relevance and importance of developing zebrafish research further and strengthening community benefit from each other's expertise.

## Advancing neuroscience through zebrafish research

The neuroscience field has leveraged the zebrafish for both fundamental biological research and disease modelling, as its genetic tractability, optical transparency and conserved brain organisation make it a powerful vertebrate model. During the sessions, Katy Marshall-Phelps (Rafael Almeida laboratory, University of Edinburgh) presented a mechanism of non-synaptic neurotransmitter release that drives neuron-glia communication in myelinated circuit formation (Marshall-Phelps and Almeida, 2024). Xinwei Wang (Tom Baden laboratory, University of Sussex) demonstrated evolutionary divergence in zebrafish retinal signalling, showing that bipolar cells use both mGluR6 and excitatory amino acid transporters for On-pathway signalling, unlike mammals. Emma Dumble (Tim Czopka laboratory, University of Edinburgh) revealed the non-canonical role of oligodendrocyte precursor cells in sculpting retinal ganglion cell arbours that extend beyond their traditional myelination function during development. Min-Kyeung Choi (Soojin Ryu laboratory, University of Exeter) shared results from single-cell multiome analysis of neuroendocrine cell clusters in the pre-optic area associated with stress response.

Keynote speaker David Lyons (University of Edinburgh) presented impressive work on the dynamics of myelin structure and repair mechanisms. His group identified a conserved mechanism of myelin repair that was first discovered in zebrafish, and his talk highlighted promising strategies for myelin repair in neurological disease studies.

## Employing zebrafish as a powerful model for toxicology

The use of zebrafish as a model for toxicology studies was emphasised throughout the meeting, demonstrating how this versatile model advances our understanding of environmental impacts on human health. Aya Takesono (Charles Tyler laboratory, University of Exeter) presented findings on the role of oestrogen and endocrine-disrupting chemicals on vertebrate brain development using a zebrafish oestrogen biosensor transgenic model (Takesono et al., 2022). Golsana Haghdousti (Karin Tuschl and Jason Rihel laboratory, UCL) presented a talk on utilising zebrafish as a model for manganese overload in parkinsonism (Alba-González et al., 2024). A research talk given by Ada Jimenez-Gonzalez (Andrew Grierson laboratory, University of Sheffield) focussed on using zebrafish to examine zinc exposure as a risk factor for amyotrophic lateral sclerosis (ALS). The study highlights the advantages of using zebrafish as a model to investigate short- and long-term effects of environmental pollutants in ALS, with broader implications that can be applied to human health.

Sylvia Dimitriadou (Charles Tyler laboratory, University of Exeter) discussed refining anaesthetic use during early zebrafish development. Efficacy of anaesthetic agents varied during early zebrafish development, underscoring the need for careful selection of anaesthetic agents to support ethical and reproducible scientific research. Importantly, the research presented aligns with the refinement aspect of the 3Rs [from the National Centre for the Replacement, Refinement and Reduction of Animals in Research (NC3Rs)] and will ultimately improve the reproducibility of experiments undertaken at the early larval stages of zebrafish development.

## Emerging technologies in zebrafish

A major strength of the zebrafish research community lies in its continuous technological innovation, and the meeting featured contributions from leading experts driving methodological advancements in the field. Yosuke Ono (Steffen Scholpp laboratory, University of Exeter) presented highly efficient prime editing approaches for precise zebrafish genome modification, demonstrating up to fourfold increases in editing efficiency compared to traditional methods (Ono et al., 2025). Additionally, David Gurevich (University of Bath) introduced novel boronic acid-fluorescent conjugates as sugar-responsive probes for glucose monitoring *in vivo* in zebrafish, opening new avenues for metabolic imaging applications.

## Beyond zebrafish

Alongside the talks using zebrafish as a model, the meeting also welcomed experts in alternative teleost models. There were talks and posters focused on the newest developments in the establishment of the African turquoise killifish (*Nothobranchius furzeri*) as a model of ageing from Ryan MacDonald's laboratory (UCL) and the Arabian killifish (*Aphanius dispar*) as a model of human disease from Tetsu Kudoh's laboratory (University of Exeter). Finally, we were delighted to welcome Miguel Concha, from Universidad de Chile, who presented the latest work from his laboratory on the behaviour of embryonic cells during epiboly of the South American annual killifish (*Austrolebias nigripinnis*).

## Zebrafish facility users' community session

Community building was a key highlight of the meeting. During the final day, five zebrafish facility managers led a series of discussions addressing current challenges faced by UK zebrafish facilities and users and proposed potential solutions. This session underscored the value of a more coordinated national infrastructure, and UKZF2025 provided an ideal platform to initiate these discussions. Chaired by Greg Paull (University of Exeter), the session began with summaries of the management structure and research support available at each facility, followed by a broader discussion on zebrafish movement between facilities, reporting and reproducibility, and the implementation of the 3Rs (Replacement, Reduction and Refinement). These discussions highlighted the importance of close collaboration between facility managers and researchers to develop fit-for-purpose solutions.

Nicola Goodwin (University of Cambridge) outlined the current constraints on zebrafish movement to and within the UK. She described the barriers that are currently restricting facility users looking to import and export fish lines and highlighted ongoing efforts by the facility managers to continue to allow the sharing of lines between institutions. Greg Paull followed with a discussion on ‘Building national capability for zebrafish research’. He proposed the development of a UK-wide database to share information on available lines in the UK zebrafish community and the establishment of national hubs that can store cryopreserved lines and export these lines around the UK and to the international zebrafish community.

The second topic ‘Reporting and reproducibility’ was presented by Claire Allen (University of Sheffield), who discussed common reproducibility issues faced by zebrafish research facilities and potentially strategies to overcome these. This included standardisation versus heterogeneity of laboratory conditions for the fish, the importance of using the Animal Research: Reporting of *In Vivo* Experiments (ARRIVE) guidelines and opportunities to expand these guidelines to better support zebrafish research. She also proposed engagement with a monthly webinar series on reporting and reproducibility to discuss practices amongst facilities and to help overcome issues.

The final presentation ‘Zebrafish genotyping methods: what is out there?’ was given by Heather Callaway (UCL) and Dave Maley (University of Exeter). They reviewed the different methods of genotyping currently used across UK facilities, discussed the application of the 3Rs to the methods used and identified barriers affecting the uptake of the different methods within the UK community. Rodrigo Young (UCL) then introduced a new database for UK zebrafish lines he had been developing called ‘FinFinder’ (finfinder.co.uk), which allows UK zebrafish facility users to upload any zebrafish lines they are willing to share with other UK facilities.

To close the community presentations, Stefan Schulte-Merker (University of Münster and President of the European Zebrafish Society) provided an update from the EU directive on zebrafish accommodation and how this, in turn, may impact the legislation for the UK. A question-and-answer discussion then took place, giving attendees the opportunity to engage directly with the speakers about the topics that were discussed.

## Take-home message

UKZF2025 successfully brought together a diverse group of zebrafish researchers, technical staff and facility managers from across the UK. The talks and poster sessions during the meeting provided an invaluable platform for early-career researchers and principal investigators to share recent and unpublished work within a supportive and collaborative community. The breadth of research showcased throughout the meeting emphasised the many strengths of zebrafish as a versatile model organism. Crucially, the meeting fostered open discussion among the UK zebrafish community about shared challenges and opportunities facing zebrafish researchers and facility managers nationwide.

Overall, UKZF2025 was a resounding success. The meeting concluded with the exciting announcement that the UK zebrafish community will reunite for the second UK zebrafish meeting in Edinburgh in 2027!
